# Identification of precision treatment strategies for relapsed/refractory multiple myeloma by functional drug sensitivity testing

**DOI:** 10.18632/oncotarget.17630

**Published:** 2017-05-05

**Authors:** Muntasir Mamun Majumder, Raija Silvennoinen, Pekka Anttila, David Tamborero, Samuli Eldfors, Bhagwan Yadav, Riikka Karjalainen, Heikki Kuusanmäki, Juha Lievonen, Alun Parsons, Minna Suvela, Esa Jantunen, Kimmo Porkka, Caroline A. Heckman

**Affiliations:** ^1^ Institute for Molecular Medicine Finland (FIMM), Helsinki Institute of Life Science, University of Helsinki, Helsinki, Finland; ^2^ Department of Medicine, Kuopio University Hospital, Kuopio, Finland; ^3^ Department of Hematology, Helsinki University Hospital, Comprehensive Cancer Center, Helsinki, Finland; ^4^ Research Unit on Biomedical Informatics, Department of Experimental and Health Sciences, University Pompeu Fabra, Barcelona, Spain; ^5^ Hematology Research Unit, University of Helsinki, Helsinki, Finland

**Keywords:** multiple myeloma, functional screening, drug sensitivity and resistance testing, precision medicine, high-risk myeloma

## Abstract

Novel agents have increased survival of multiple myeloma (MM) patients, however high-risk and relapsed/refractory patients remain challenging to treat and their outcome is poor. To identify novel therapies and aid treatment selection for MM, we assessed the *ex vivo* sensitivity of 50 MM patient samples to 308 approved and investigational drugs. With the results we i) classified patients based on their *ex vivo* drug response profile; ii) identified and matched potential drug candidates to recurrent cytogenetic alterations; and iii) correlated *ex vivo* drug sensitivity to patient outcome. Based on their drug sensitivity profiles, MM patients were stratified into four distinct subgroups with varied survival outcomes. Patients with progressive disease and poor survival clustered in a drug response group exhibiting high sensitivity to signal transduction inhibitors. Del(17p) positive samples were resistant to most drugs tested with the exception of histone deacetylase and BCL2 inhibitors. Samples positive for t(4;14) were highly sensitive to immunomodulatory drugs, proteasome inhibitors and several targeted drugs. Three patients treated based on the *ex vivo* results showed good response to the selected treatments. Our results demonstrate that *ex vivo* drug testing may potentially be applied to optimize treatment selection and achieve therapeutic benefit for relapsed/refractory MM.

## INTRODUCTION

Immunomodulatory drugs and proteasome inhibitors combined with alkylating agents and steroids have improved the outcome of MM patients [1]. While some patients experience long remission, prognosis is still poor for high-risk patients. Heterogeneity in treatment response may be influenced by several patient or disease related features such as frailty, age, comorbidity, clinical stage and the presence of one or more cytogenetic abnormalities [2]. Patients may exhibit both *de novo* or acquired resistance to current therapies by mechanisms (i.e. clonal heterogeneity and evolution) that are still poorly understood [3]. Prior information on responses to approved myeloma and other oncology drugs remains crucial to determine the timing and sequence of treatments. With the recent exception of venetoclax in BCL-2 driven t(11;14) MM [4], genomically guided treatments have not been successful in MM. Other targeted agents such as mTOR and HDAC inhibitors have been clinically investigated, however, these were not biomarker driven studies and thus not targeted to patients likely to respond [5–10]. A real time and viable means of assessing drug response using the patient’s own malignant cells could accelerate the design of individualized treatment strategies and improve outcome.

The myeloma genome contains complex cytogenetic alterations that affect both the number and structure of chromosomes [11]. Recurrent cytogenetic alterations are well recognized as biomarkers defining treatment outcome and prognosis. Based on the presence or absence of these alterations myeloma has been stratified into high-risk (HR), standard-risk (SR) and low-risk (LR) groups, with del(17p) and in general t(4;14) indicating the worst prognosis among the cytogenetic aberrations [12–14]. New drugs and therapeutic innovations are urgently needed for HR patients who comprise 20% of the myeloma population and have a median overall survival of only two years. Furthermore, patients who are refractory to both bortezomib and lenalidomide have very poor outcome with median survival of only nine months [15]. Several efforts have elucidated the genomic landscape in myeloma [16–22], albeit systematic analysis linking cytogenetic alterations to drug response is lacking.

Increasing genomic complexity during disease progression leads to the activation of multiple signaling pathways that are known to contribute to treatment resistance, which can potentially be targeted using signal transduction inhibitors in combination with approved drugs. However, it is extremely important to identify the responding patients in advance to apply those drugs to maximize benefit. Here, we describe *ex vivo* drug sensitivity and resistance profiling of 50 MM patient samples to 308 drugs and use the overall drug response profile to classify patients. We compared the responses to the patient cytogenetics to identify potential drug candidates for each karyotype and identify novel treatment strategies for relapsed/refractory (RR) MM. Results were used to tailor treatment for three patients. Our studies indicate that an individualized approach by functional, *ex vivo* drug testing may be effectively applied to MM to provide additional information to guide treatment selection and potentially improve therapeutic benefit.

## RESULTS

### Myeloma patients can be stratified based on distinct drug sensitivity profiles

**Figure 1:**
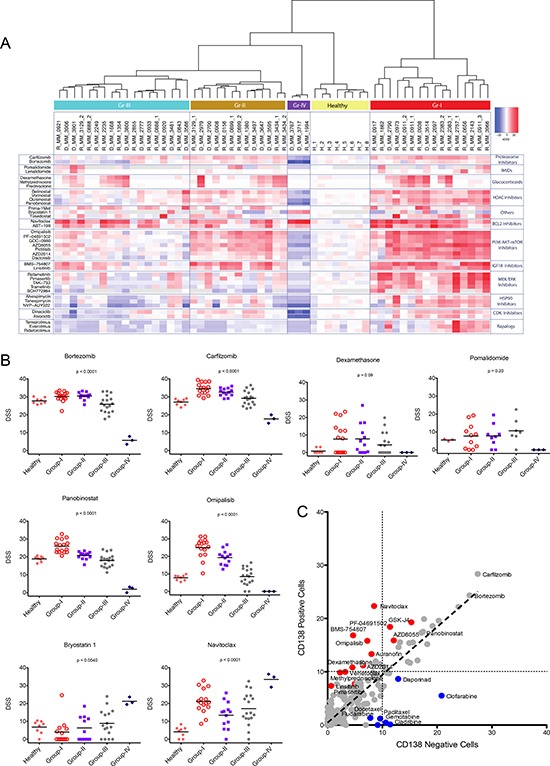
*Ex vivo* drug sensitivity profiling results in stratification of MM patients in four chemosensitivity subgroups (**A**) Summary view of clustering analysis of myeloma patients based on their overall *ex vivo* drug sensitivity. The distinct drug response patterns results in four taxonomic groups. Columns represent samples and rows represent drugs. The data summarize the selective drug sensitivity scores (sDSS) of the samples to the drugs. Detailed heatmaps are shown in Supplementary Figures 1 and 2. Bootstrap analysis to show stability of clustering is shown in Supplementary Figure 3. (**B**) *Ex vivo* responses (DSS) by group to a selection of approved and investigational drugs. Graphs comparing IC50 for the same drugs are presented in Supplementary Figure 4B. (**C**) Comparison of mean *ex vivo* responses (DSS) to all tested drugs in paired CD138+ and CD138- cells for 9 individual MM patients. Red indicates drugs that show better effect to CD138+ cells. Blue indicates drugs that target CD138- cells. Correlation plots for individual samples are shown in Supplementary Figure 5.

To assess drug efficacy and compare the drug response data across patient samples we used a quantitative drug sensitivity score (DSS). DSS is a modified form of the area under the curve (AUC) calculation that integrates multiple dose response parameters for each drug, while a selective drug sensitivity score (sDSS) was calculated by taking into account the sensitivity of healthy BM control samples (*n* = 8) to the drugs and subtracting the mean DSS of the controls from the DSS of the tumor sample [23, 24]. Unsupervised hierarchical clustering of the DSS and sDSS for each patient identified four distinct patient groups (I-IV) based on their sensitivity to 308 drugs (summary of molecules of interest and sDSS of samples in Figure 1A and all results shown in Supplementary Figures 1 and 2). Conventional chemotherapeutics and proteasome inhibitors were relatively non-selective when comparing the responses to these drugs between myeloma patient and healthy donor cells. However, the patient samples showed varied responses to targeted agents including many signal transduction inhibitors across the different chemosensitivity groups. Samples in group I (*n* = 16) showed selective sensitivity to several signal transduction inhibitors including those targeting IGF1R-PI3K-mTOR, HDAC, MEK, CDK and HSP90 (Figure 1B and Supplementary Figure 4A). Although group II samples (*n* = 13) responded to many of the same drugs, the sensitivities were more moderate, in particular to MEK, HDAC and HSP90 inhibitors. In addition, group II samples lacked sensitivity to rapalogs. In contrast, group III samples (*n* = 18) were relatively insensitive to many targeted therapies and exhibited diminished response to most drugs compared to healthy controls. The most striking response was observed for group IV (*n* = 3). Although the number of samples comprising this group was small, the samples were distinctively resistant to almost all drugs tested with the exception of bryostatin1 and the pan-BCL2 inhibitor navitoclax (Figure 1A and 1B). In addition, BCL2 inhibitors were active across all four chemosensitivity groups (Figure 1A).

### Signal transduction inhibitors selectively target multiple myeloma cells

To determine if the drug responses were specific for CD138+ cells, we tested separately CD138+ cells and the remaining BM-MNCs (CD138-) using samples from nine patients. Supplementary Table 1 CD138- cells exhibited little sensitivity to signal transduction inhibitors targeting PI3K-AKT-mTOR, MAPK, and IGF1R, HSP90 and BCL2 family members, with responses similar to that observed in BM cells from healthy individuals. In contrast, MM derived CD138+ cells were insensitive to nucleoside analogues including clofarabine, cladribine and gemcitabine, while CD138- cells were sensitive (Figure 1C). The drug sensitivity pattern of MM CD138+ cells suggests dependence on specific signaling pathways known to be pathogenic in MM and which can potentially be therapeutically exploited.

### Acquired sensitivity to targeted therapies predicts poor survival

**Figure 2:**
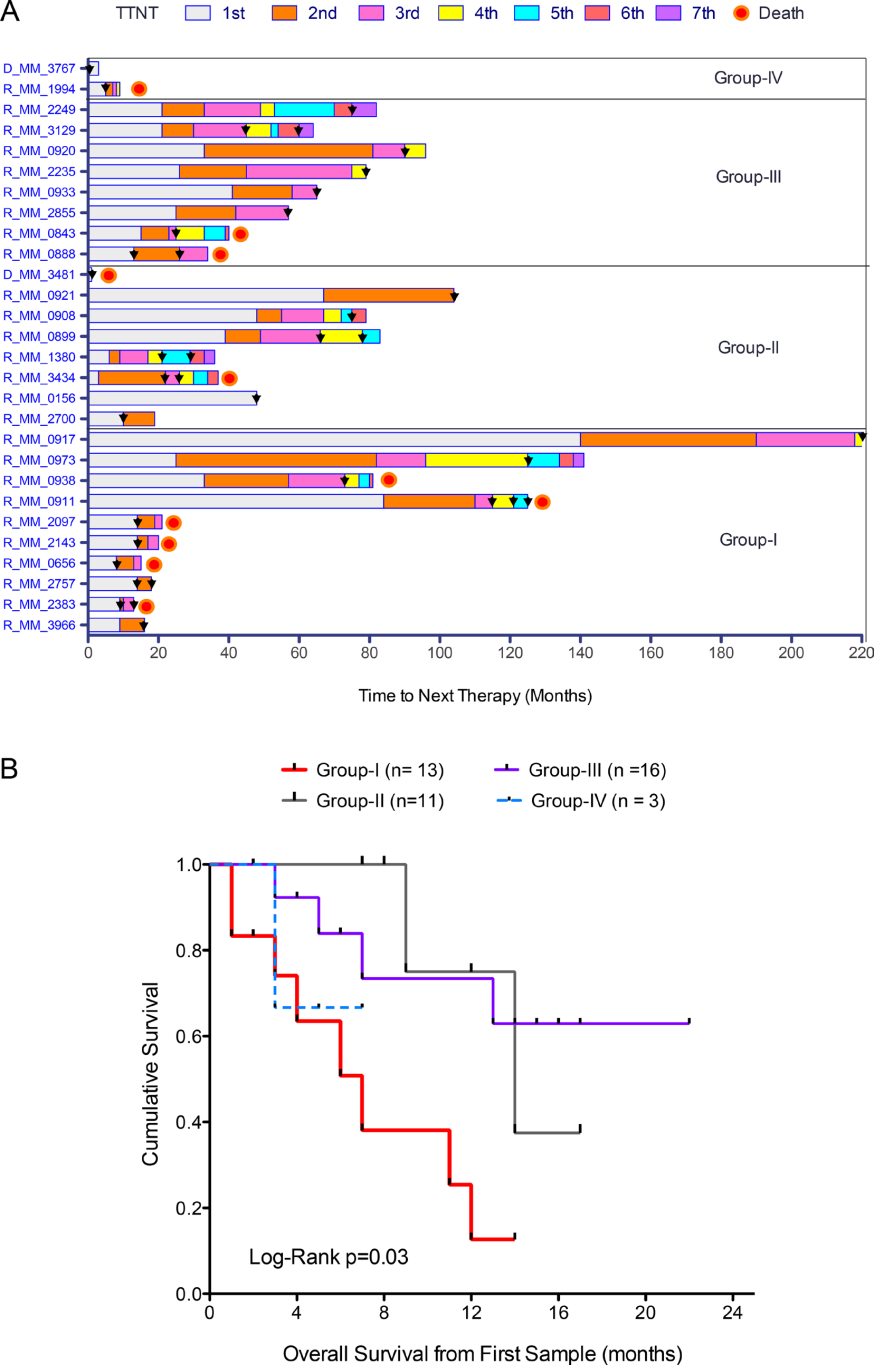
Drug sensitivity stratification predicts disease progression and overall survival (**A**) Time to next treatment (TTNT) for relapsed patients (*n* = 27) and patients at diagnosis who had relapsed (*n* = 1) from the four different chemosensitivity groups. Colored bar sections represent the different lines of treatment and black arrowheads indicate sampling time for *ex vivo* drug testing. (**B**) Kaplan-Meier graph showing significant differences in overall survival of the patients comprising the four chemosensitivity groups.

To assess the value of the drug sensitivity results in predicting patient outcome, we compared time to next therapy (TTNT) among the four different chemosensitivity groups. While most of the samples analyzed came from relapsed/refractory patients with expected poor outcome, TTNT differed between the four groups (Figure 2A). Interestingly, patients comprising the most sensitive group to signal transduction inhibitors (Group I) had progressive disease with very short TTNT and overall survival (Figure 2A and 2B) with a hazard ratio of 4.66 (CI95% 1.71–12.77). Patients comprising groups II and III exhibited similar TTNT, and although group IV patients were too few to evaluate, these patients showed short treatment response followed by progression.

### Correlation with cytogenetic alterations reveals novel treatment options for high-risk myeloma patients

**Figure 3:**
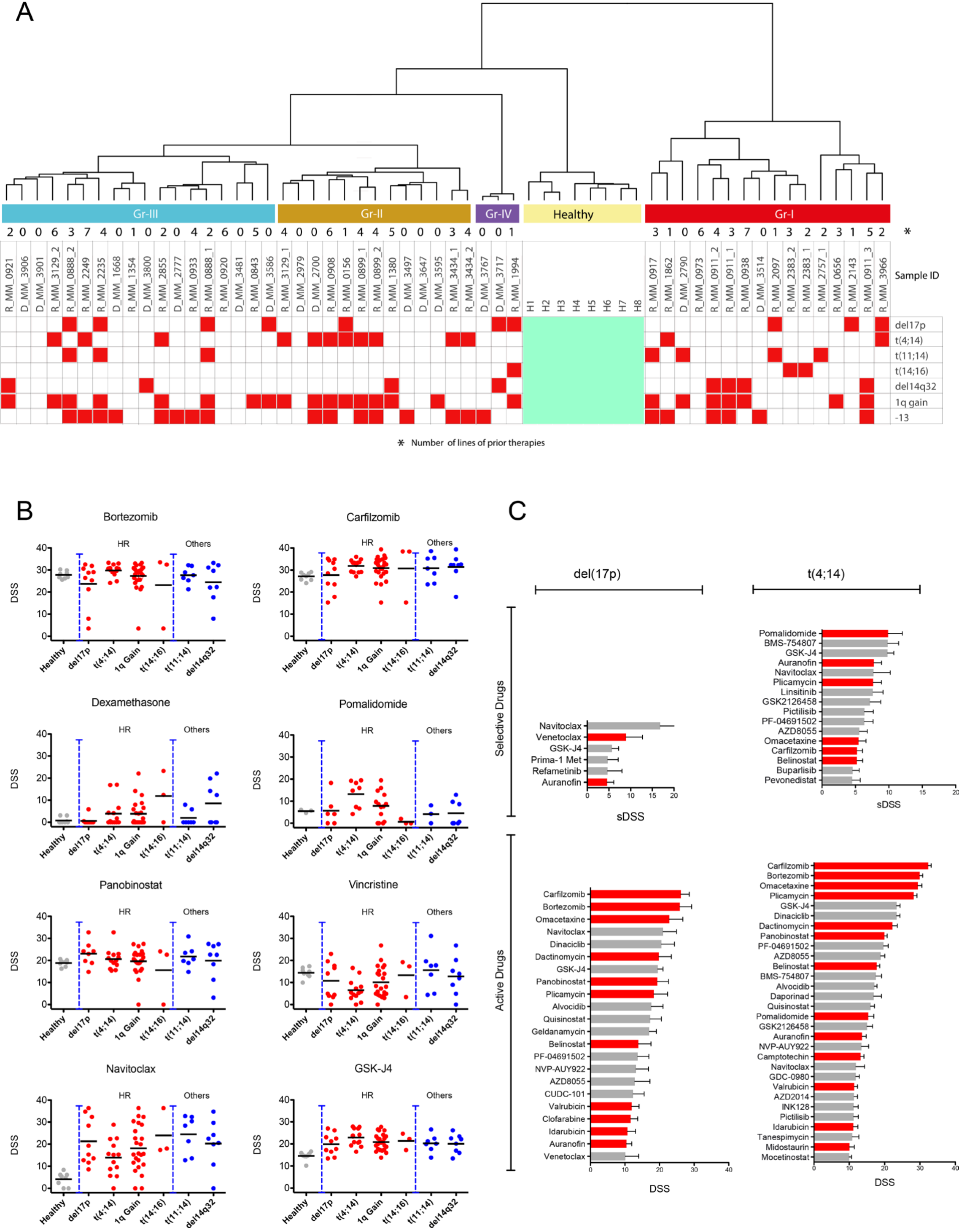
Cytogenetic markers of the chemosensitivity groups and top scoring drugs for high-risk patients (**A**) Cytogenetic markers for the drug-tested patients in relation to the chemosensitivity groups. Samples from patients with del(17p) were predominantly in groups III and IV, while the majority of t(4;14) patient samples were in group II. (**B**) *Ex vivo* drug responses to standard of care and recently approved drugs as well as the investigational drug navitoclax and histone demethylase inhibitor GSK-J4 subdivided by specific cytogenetic alterations (del(17p), *n* = 10; t(4;14), *n* = 13; t(11;14), *n* = 7; +1q, *n* = 24; t(14;16), *n* = 3; del14q32, *n* = 7). Graphs using IC50 for the same drugs are presented in Supplementary Figure 6B. (**C**) Top scoring selective inhibitors for del(17p) and t(4;14) patients (top bar plots), and the most active inhibitors (lower bar plots) with approved drugs indicated in red.

Myeloma cells contain numerous and complex genetic alterations with several karyotypes of prognostic importance including those for HR patients such as del(17p), t(4;14), t(14;16) and 1q gain. We investigated if there was correlation between drug response profiles and karyotypes. Samples from patients with t(4;14) predominantly clustered in group II (*n* = 8/13), but were also present in group III (*n* = 3/18) and group I (*n* = 2/16) (Figure 3A), while samples from patients with del(17p) were present in all groups, but predominant in the more resistant groups III (*n* = 4/18) and IV (*n* = 2/3) (Figure 3A). Samples from two patients with both del(17p) and t(4;14) clustered in groups I and II.

Del(17p) patient samples tended to be very resistant with some exceptions. For example, del(17p) along with other HR patient samples exhibited good sensitivity to panobinostat (Figure 3B). The del(17p) cells were also sensitive to pan-BCL2 inhibitor navitoclax (Figure 3B and 3C) and modestly to specific BCL2 inhibitor venetoclax, although samples from patients with other karyotypes also showed similar sensitivity (Figure 3C). The highest sensitivity to venetoclax was observed in samples from patients with t(11;14) and t(14;16) (Supplementary Figure 6A). The del(17p) samples were sensitive to several other drugs, but these drugs were less selective and showed similar activity against healthy BM cells (Figure 3B and 3C).

In contrast, t(4;14) cells were less sensitive to navitoclax, but were highly sensitive to pomalidomide (Figure 3B and 3C). The t(4;14) samples were also sensitive to proteasome inhibitors, although the activity of these drugs tended to be less selective in the assay. Other drugs with activity against t(4;14) samples were IGF1R (BMS-754807 and linsitinib) and dual PI3K-mTOR inhibitors (GSK2126458, PF-04691502), suggesting potential activity of these signaling molecules in t(4;14) cells (Supplementary Figure 6A). In addition, t(4;14) samples were sensitive to GSK-J4, an inhibitor of the histone lysine demethylase JMJD3/KDM6B (Figure 3C). Interestingly, FGFR inhibitors such as dovitinib and NVP-BGJ398 lacked efficacy towards t(4;14) cells although *FGFR3* was highly expressed in these samples (Supplementary Figure 7).

### *Ex vivo*-*in vivo* correlation of drug sensitivity

**Figure 4:**
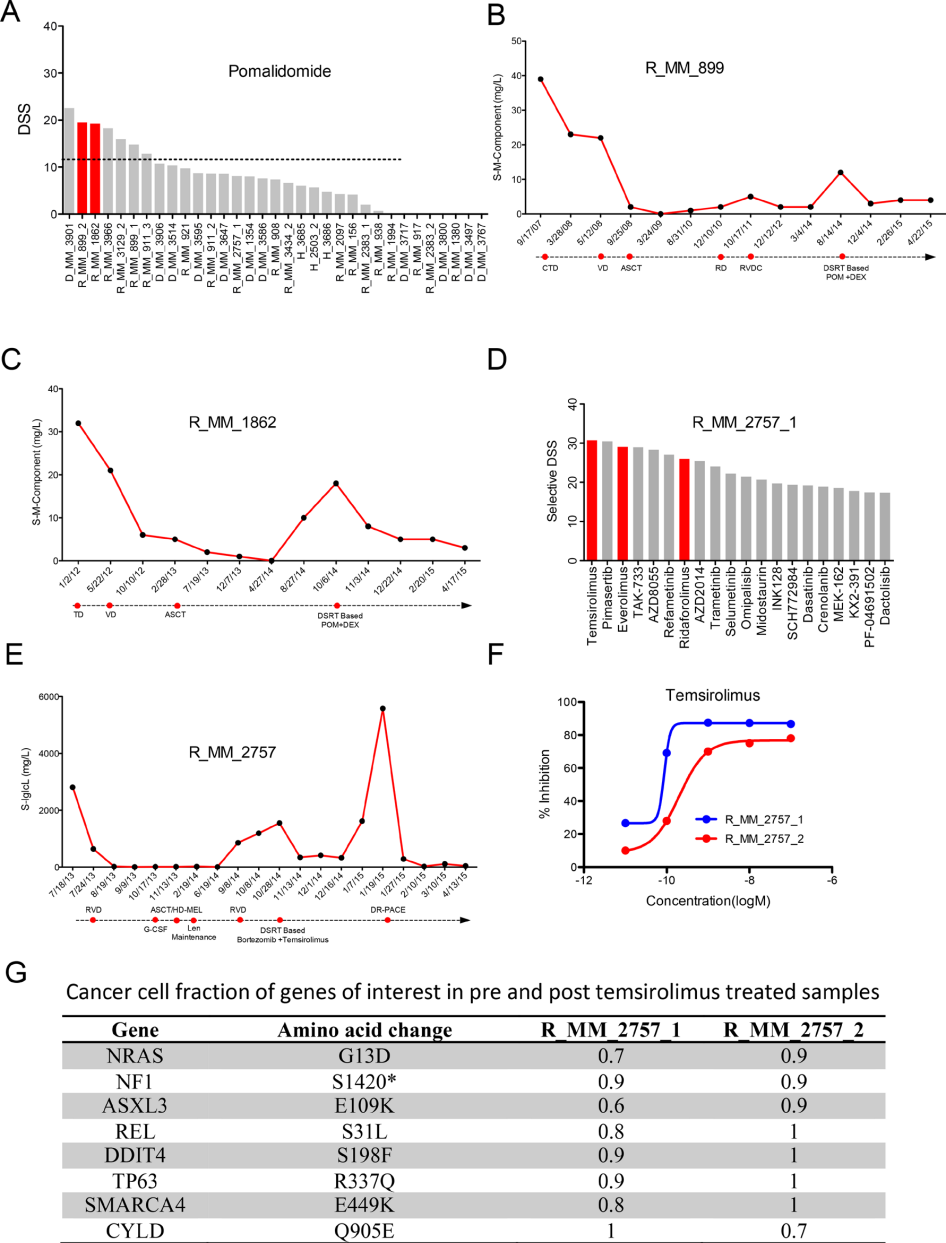
*Ex vivo* – *in vivo* correlation of drug response (**A**) The waterfall plot ranks the patients based on *ex vivo* sensitivity to pomalidomide, with two patients treated with pomalidomide based on the drug sensitivity results highlighted in red. (**B** and **C**) Responses to pomalidomide and earlier lines of treatment based on serum M component level for the two patients R_MM_899 and R_MM_1862. (**D**) The most selective drugs for the first sample from patient R_MM_2757_1 tested in the *ex vivo* assay. Top scoring selective drugs included rapalogs temsirolimus, everolimus and ridaforolimus highlighted in red. (**E**) Response of the R_MM_2757 patient to different lines of treatment including the combination of temsirolimus and bortezomib as measured by serum Iglcλ level. (**F**) *Ex vivo* sensitivity of CD138+ cells from the R_MM_2757 patient pre- (blue line) and post-temsirolimus/bortezomib treatment (red line) with a shift in dose response curve indicating acquired resistance. (**G**) Cancer cell fractions for (CCF) of somatic alterations to genes of interest in the pre- (R_MM_2757_1) and post-temsirolimus (R_MM_2757_2) treatment samples from patient R_MM_2757.

For three patients with advanced stage disease, treatment was decided based on the drug testing results. Two t(4;14) positive relapsed patients showing extremely good *ex vivo* sensitivity to pomalidomide (Figure 4A) were treated with a combination of pomalidomide and dexamethasone. Pomalidomide was used at 4 mg/day on days 1-21 and dexamethasone 40 mg weekly of each 28-day cycle. The combination resulted in minimal response for 32 weeks with eight cycles for the first patient and partial response for 16 weeks with four cycles for the second patient (Figure 4B and 4C). Oral cyclophosphamide 450 mg weekly was added to the latter patient resulting in sustained partial response after six cycles, 24 weeks. Lack of *ex vivo* sensitivity to dexamethasone (Supplementary Figure 8) suggested that pomalidomide and not dexamethasone had a direct anti-tumor effect *in vivo*.

For a t(11;14) patient, drug testing analysis at the time of relapse showed exceptional sensitivity to rapalogs (temsirolimus, everolimus and ridaforolimus) (Figure 4D). Prior to testing, the patient had previously received three cycles of bortezomib, lenalidomide and dexamethasone followed by a single autologous stem cell transplant and lenalidomide maintenance resulting in stringent complete remission for 11 months. *Ex vivo* drug testing showed that the cells were sensitive to bortezomib, modestly sensitive to immunomodulatory drugs, but insensitive to dexamethasone (Supplementary Figure 9). Based on a phase II study, the patient was treated with a combination of bortezomib and temsirolimus (bortezomib 1.6 mg/m^2^ on days 1, 8, 15 and 22 and temsirolimus 25 mg on days 1, 8, 15, 22, 29 for 35-day cycles) [6, 25]. The patient experienced a dramatic response with rapid reduction of serum free light chain lambda from 1550 mg/L to 343 mg/L in two weeks. Progression free survival of 84 days was achieved before the second progression (Figure 4E). At this point another BM sample was taken and drug sensitivity assessed. An overall decrease in drug response was seen with loss of sensitivity to temsirolimus compared to the previous sample (Figure 4F). Exome sequence analysis of the samples showed small changes in the clonal composition between pre- and post-temsirolimus treatment (Figure 4G). The Ras pathway, which is upstream of mTOR, was likely activated by mutations to *NRAS* and *NF1*, with the *NRAS* mutation and a *REL* mutation enriched in the post-temsirolimus treatment sample. In addition, a mutation to DNA-Damage-Inducible Transcript 4 (*DDIT4*), a repressor of mTORC1, was also detected in both samples [26–28]. These events collectively could render the cells dependent on mTOR signaling as indicated by the sensitivity to rapalogs, while mutations to *NRAS* and *NF1* may have also contributed to the sensitivity to MEK inhibitors (e.g. pimasertib, TAK-733, refametinib, trametinib, selumetinib).

## DISCUSSION

Considering both genetics and clinical outcome, multiple myeloma presents extreme inter- and intra-individual heterogeneity [21] resulting in varied treatment response and highlighting the importance of defining patient populations prior to treatment with targeted therapies. In this study we showed that this variation is also observed through comprehensive drug sensitivity profiling and patients could be classified into different chemosensitive groups based on their *ex vivo* drug response profiles. Surprisingly, correlating the groups to patient outcome showed that patients comprising the most highly sensitive group (group I) had the shortest survival. As inferred from susceptibility to numerous signal transduction inhibitors, the activation of multiple signaling pathways in the malignant cells may drive the disease of these patients and potentially be the cause of relapse and acquired resistance to approved therapies. Noticeably, many group I samples were from standard-risk patients at relapse and end stage disease. While the samples exhibited sensitivity to many current therapies, multiple escape routes could circumvent blockade on growth. These patients could potentially benefit from treatment strategies based on signal transduction inhibitors combined with other standard therapies.

Among standard of care drugs, there was considerable variation in response to glucocorticoids. Dexamethasone, methylprednisolone and prednisolone showed similar patterns in efficacy with clearly responsive and non-responsive samples. Further analysis of mutation and gene expression profiles is needed to identify potential indicators of response, such as mutations to the glucocorticoid receptor or specific gene expression signatures [29, 30]. Emerging studies are providing a much better understanding of the impact of therapy on the clonal architecture and evolution of the disease [31, 32]. Although exome sequence analysis of samples taken before and after bortezomib/temsirolimus treatment of one patient did not reveal any newly acquired mutations, the clonal cell fractions differed between samples indicating that even targeted treatments can have an impact on clonal architecture. Nevertheless, knowledge of the drug sensitivity landscape by *ex vivo* testing before treatment can potentially be exploited for patients who are more refractory to currently used therapies and may benefit from tailored treatment, while molecular profiling will be helpful to understand changes induced by treatment and identify indicators of response.

By correlating standard cytogenetic markers with drug response profiles, we evaluated if any genetically driven treatment options were available, particularly for high-risk patients. Cells with t(4;14) were sensitive to proteasome inhibitors and immunomodulatory drugs. This is supported by findings from a clinical trial suggesting that high-risk patients with t(4;14) are likely to benefit from receiving these drugs as frontline treatment [33]. The t(4;14) leads to overexpression of *FGFR3* and *MMSET* [34]. Although we observed enhanced expression of *FGFR3* in the t(4;14) samples, the cells were not sensitive to FGFR inhibitors (e.g. dovitinib, NVP-BGJ398). Efficacy of FGFR3 inhibitors has been explored in MM and other indications with FGFR1-3 amplifications or somatic aberrations [35–37]. However, single agent activity is modest and often independent of FGFR status. *MMSET* encodes the histone methyltransferase NSD2 [38]. Deregulation of *MMSET* is associated with a global increase in dimethylation of lysine 36 on histone H3 (H3K36me2) and simultaneous decrease in lysine 27 trimethylation on histone H3 (H3K27me3), which is a repressive histone modification [29, 30]. We found that t(4;14) cells were sensitive to GSK-J4, an inhibitor of the histone demethylase JMJD3/KDM6B, which has H3K27me3 as a substrate. For del(17p) samples, the drug response profiles correlated to the clinical scenario with the cells resistant to most drugs tested. There were few drugs that showed selective activity towards del(17p) cells. However, the cells tended to be more sensitive to panobinostat and relapsed del(17p) patients could potentially benefit from this drug. The del(17p) cells were also sensitive to the BCL2/BCL-xL inhibitor navitoclax, and modestly sensitive to specific BCL2 inhibitor venetoclax, suggesting that anti-apoptotic factors such as BCL-xL may be important for del(17p) cell survival. Our results with BCL2 inhibitors together with earlier studies [39–41] further emphasize the need for clinical investigations with these drugs.

Comparison of drug responses between MM CD138+, MM CD138- and healthy BM cells allowed us to distinguish between malignant and non-malignant responses, and identify drugs with maximum efficacy against MM plasma cells and minimal activity towards healthy cell populations. Our results demonstrate that sensitivity to several signal transduction inhibitors could be observed in the MM plasma cell fraction, whereas the response in the non-plasma cell fraction was similar to that of healthy individuals. These results emphasize that molecular alterations present in malignant plasma cells may be selectively exploited by targeted therapies thereby minimizing treatment related toxicities to other cell populations. Although the platform was useful at determining drug responses on the tested cell population, it will be important to develop the assay further to take into account the effects of the microenvironment on drug response. While the supportive culture medium was from a bone marrow stromal cell line, the assay could not account for cell adhesion mediated drug resistance or the impact of hypoxia on drug response. These could be assessed using for example cell co-cultures [42] and hypoxia chambers. Furthermore, it will be important to ascertain indirect tumor cytotoxicity of the drugs through stimulation of other immune cells by immunomodulatory drugs. Nonetheless, the current assay provides a means to quickly assess the impact of hundreds of drugs at several concentrations on individual patient samples.

Guiding treatment decisions based on personalized drug sensitivity testing is compelling and has not been widely investigated. We observed exceptional sensitivity to rapalogs for one patient and to pomalidomide for two other patients in the *ex vivo* assay, accompanied by very clear *in vivo* response. Our results show that comprehensive, functional, drug sensitivity assessment applied to MM patients provides information that can be used to understand variability in drug response and to classify patients based on their chemosensitivity profile. Importantly, we were able to identify candidate drugs that may be effective for treating HR and RRMM patients and the assay provided informative results to guide treatment selection. By using a panel of signal transduction inhibitors, we were able to identify active pathways in the MM cells targetable by these drugs and which can be further investigated for drug development. As more drugs are approved for MM, determining the best treatment and timing for each patient becomes more challenging. *Ex vivo* testing may therefore be clinically useful for therapy guidance.

## MATERIALS AND METHODS

### Patients and samples

The ethics committees of the participating hospitals approved the study with patient and healthy donor samples obtained following informed consent in compliance with the Declaration of Helsinki. A total of 58 bone marrow (BM) aspirates were collected from 16 diagnostic and 27 relapse patients and 8 healthy donors. Patient characteristics are detailed in Supplementary Table 2. No exclusion criteria were applied to the patients and the samples were collected prospectively. Data collection was continued at successive relapses to follow disease progression.

### Cytogenetics

Plasma cells were selected by immunomagnetic bead enrichment of CD138+ cells (Human Whole Blood CD138 Microbeads Column kit, Miltenyi Biotec, Bergisch Gladbach, Germany). Selected cells (*n* ≥ 100) were used for interphase fluorescence *in situ* hybridization (FISH) following the guidelines of the European Myeloma Network 2012 [43]. FISH probes are listed in Supplementary Table 3.

### Drug sensitivity and resistance testing

CD138+ cells were enriched using the EasySep™ Human CD138 Positive Selection kit (StemCell Technologies, Grenoble, France) from the mononuclear cell fraction of BM aspirates following gradient separation (Ficoll-Paque PREMIUM; GE Healthcare, Little Chalfont, Buckinghamshire, UK). Drug sensitivity and resistance testing (DSRT) was performed based on methods described previously [23]. CD138+ cells derived from myeloma patients were tested against 308 compounds at 5 concentrations in 10-fold dilutions covering a 10,000-fold concentration range (1–10,000 nM). The drug panel included approved oncology drugs (*n* = 141) and investigational compounds (*n* = 167) targeting multiple signaling networks and molecular targets (Supplementary Table 4). In brief, 5μl of cell culture medium comprised of RPMI 1640 medium supplemented with 10% fetal bovine serum, 2 mM L-glutamine, penicillin (100 U/ml), streptomycin (100 μg/ml) and 25% conditioned medium from the HS-5 human BM stromal cell line was added to 384 well drug plates and shaken for 5 min to dissolve the compounds. CD138+ cells were diluted in the culture medium and 20μl of the cell suspension containing 5000 cells was transferred to each well using a MultiDrop Combi peristaltic dispenser (Thermo Scientific, Waltham, MA, USA). The plates were incubated in a humidified environment at 37°C and 5% CO_2_. Cell viability was measured after 72 h using the CellTiter-Glo assay (Promega, Madison, WI, USA) with a PHERAstar® microplate reader (BMG-Labtech, Offenburg, Germany) to measure luminescence. The mean viability of untreated cells at day three was 124 ± 10.40%. The data was normalized to negative (DMSO only) and positive control wells (containing 100 μM benzethonium chloride).

### *Ex vivo* drug sensitivity data analysis

Output from the plate reader was used as input for Dotmatics software (Dotmatics Ltd, Bishops Stortford, Herts, UK) to generate dose response curves of individual drugs. A four parameter (maximum and minimum response, slope and IC50) logistic regression was applied to fit the dose response curves. Curve fitting parameters were further used to quantitate drug responses with a drug sensitivity score (DSS) as described previously [24]. DSS is a modified form of the area under the curve calculation that takes into account all four curve fitting parameters of a non linear response model, generating a single response metric that was used for downstream analyses. Higher DSS corresponds to higher sensitivity. Selective drug sensitivity scores (sDSS) indicating tumor specific sensitivity to the drugs were calculated by subtracting the mean DSS values obtained by testing BM cells from eight healthy individuals from the DSS values of the patient samples. To evaluate the relatedness of drug response profiles across all myeloma samples, we performed unsupervised hierarchical ward linkage clustering using Spearman correlation and Euclidean distance measures of the drug and sample profiles, respectively. Robustness and reproducibility of the identified subgroups/clusters were evaluated by resampling (*n* = 1000) using a bootstrapping method with Pvclust R-package [44].

### Exome sequencing

Genomic DNA was isolated from a skin biopsy and CD138+ cells using the DNeasy Blood & Tissue kit or AllPrep DNA/RNA/miRNA Universal kit (Qiagen, Hilden, Germany). Exome capture was performed using the SureSelect Clinical Research Exome kit or the SureSelect Human All Exon V5 kit (Agilent Technologies, Santa Clara, CA, USA). Sequencing was performed on HiSeq 1500 and 2500 instruments (Illumina, San Diego, CA, USA). For the skin germline control 4 × 10^7^ and 10×10^7^ 2×100 bp paired-end reads were sequenced for the skin germline control and CD138+ cells, respectively. Somatic mutations were identified and annotated as described earlier [45]. The cancer cell fraction has been calculated by using the variant allele frequency corrected by the gene ploidy and the estimated tumor purity.

### Statistical analyses

Statistical analyses were performed using GraphPad Prism version 5.0 for Mac OS, (GraphPad Software, La Jolla, California, USA) and SPSS version 23.0 Software (IBM, Endicott, New York, USA). One-way ANOVA followed by Tukey’s multiple comparisons test was performed to test variances among different groups in the scatterplots. Univariate survival analysis was carried out using the Kaplan-Meier method with log-rank test and survival curves presented. Multivariate survival analysis was performed with the Cox proportional hazard model with DSRT group, age, gender, paraprotein subtype, HR cytogenetics and clinical treatment sensitivity used as predictors. Variable selection was performed with the forward stepwise method. Results from multivariate survival analysis were presented with hazard ratios and 95% confidence intervals. In this study, the statistical significance level was a *p*-value under 0.05.

## SUPPLEMENTARY MATERIALS FIGURES AND TABLES





## Abbreviations

HRMM: high-risk multiple myeloma
RRMM: relapsed/refractory multiple myeloma
TTNT: time to next therapy
BM: bone marrow
DSS: drug sensitivity score
sDSS: selective drug sensitivity score
AUC: area under the curve
FISH: fluorescence *in situ* hybridization
