# Supplementary material for: Identification of precision treatment strategies for relapsed/refractory multiple myeloma by functional drug sensitivity testing
Source: Oncotarget. 2017 May 5;8(34):56338–50. doi: 10.18632/oncotarget.17630 (PMC5593565; doi:10.18632/oncotarget.17630)
Supplement: Supplementary file 1 [file oncotarget-08-56338-s001.pdf]

Identification of precision treatment strategies for relapsed/refractory multiple myeloma by functional drug sensitivity testing

Supplementary Materials

Supplementary Table 1: Viability of CD138+ and CD138- cell fractions at day 3

Sample ID	CD138+ (Plasma Cell)	Rest of the BM (CD138-)	DSRT response group
R_MM_1994	114	226	IV
R_MM_911_2	70	170	I
R_MM_2235	60	158	III
D_MM_3497	159	180	II
D_MM_3595	75	120	III
D_MM_3717	102	228	IV
D_MM_3647	22	120	II
R_MM_3434_1	246	180	II
R_MM_3434_2	210	Not measured	II

Supplementary Table 2: Patient characteristics

	Newly diagnosed $N = 16$	Relapsed, refractory $N = 27$
**Age, y, median (range)**	63 (51−72)	67 (52−82)
**Gender, M/F, *n* (%)**	10 (63) / 6 (37)	17 (63)/10 (37)
**Paraprotein, *n* (%)**		
**IgG-κ**	6 (38)	11 (41)
**IgG-λ**	1 (6)	3 (11)
**IgA-κ**	3 (19)	2 (7)
**IgA-λ**	2 (13)	6 (22)
**Light chain κ**	4 (25)	2 (7)
**Light chain λ**	0	3 (11)
**ISS, *n* (%)**		
**1**	4 (25)	8 (30)
**2**	8 (50)	5 (18)
**3**	3 (19	6 (22)
**Missing**	1 (6)	8 (30)
**Clonal plasma cells of all plasma cells, %, median (range)**	99.8 (97−100)	99.9 (80−100)
**Cytogenetics, *n* (%)**		
**t(4;14)**	1 (6)	9 (33)*
**Del 17p**	2 (13)	7 (26)
**No t(4;14) or del 17p**	13 (81)	15 (56)
**No. of previous treatment lines, median (range)**	0	3 (1−7)

**Treatment history of relapsed, refractory patients**			
**Treatment**	Exposed (%)	Refractory (%)	Not exposed (%)
**Alkylating agents**	59	41	0
**Lenalidomide**	15	67	18
**Bortezomib**	37	52	11

*2 patients had both t(4;14) and del 17p.

Supplementary Table 3: List of interphase FISH probes

iFISH PROBE	Kuopio University Hospital		Helsinki University Hospital	
**14q32 ; IF POSITIVE**	IGH Breakapart, LPH	Cytocell, UK	Vysis LSI IGH DC Break Apart Rearrangement Probe 30-191019	Abbott, USA
**IGH-CCND1/MYEOV**	Vysis IGH/CCND1 XT DF FISH Probe Kit, 05N33-020	Abbott, Illinois, USA	Vysis LSI IGH/CCND1 DC DF Translocation Probe, 30-191017	Abbott, USA
**IGH-FGFR3**	Vysis IGH/FGFR3 DF FISH Probe Kit – CE, 01N69-020	Abbott, Illinois, USA	Vysis LSI IGH/FGFR3 DC DF Translocation Probe, 30-191023	Abbott, USA
**IGH-MAF/WWOX**	XL t(14;16), D-5072-100-OG	Metasystems, Germany	Vysis LSI IGH/MAF DC DF 30-231014	Abbott, USA
**IGH/MAFB**	XL IGH/MAFB, D-5051-100-OG	Metasystems, Germany	XL IGH/MAFB, D-5051-100-OG	Metasystems, Germany
**IGH-CCND3**	IGH/CCND3 Translocation, Dual Fusion, LPH 040	Cytocell, UK	IGH/CCND3 Translocation LPH-040-S	Cytocell, UK
**13q14, 13q34**	Vysis D13s319/13q34 FISH Probe Kit – CE, 05N37-020	Abbott, Illinois, USA	Vysis LSI D13S319SO/13q34 30-191024	Abbott, USA
**17p13**	P53 (TP53) Deletion, LPH 017	Cytocell, UK	Vysis LSI ATM SG/p53 SO 30-191025	Abbott, USA
**1p32, 1q21**	XL 1p32/1q21, D-5049-100-OG	Metasystems, Germany	1p32.3/1q21.3 CKS1B/CDKN2C LPH 039	Cytocell, UK
**6q23**	MYB Deletion, LPH 016	Cytocell, UK	Not automatically done	
**11**	Not automatically done		Vysis LSI ATM SG/p53 SO 30-191025	Abbott, USA
**+12**	Not automatically done		Vysis LSI D13S319SO/13q34/ CEP12 30-191024	Abbott, USA
**+5/+9/+15**	Not automatically done		Vysis, LSI D5S23/S721 SG/ CEP9 SA /CEP15 SO 30-231021	Abbott, USA

Supplementary Table 4: Small molecule inhibitors used in the drug sensitivity assay (provided in a separate file). See Supplementary_Table_4

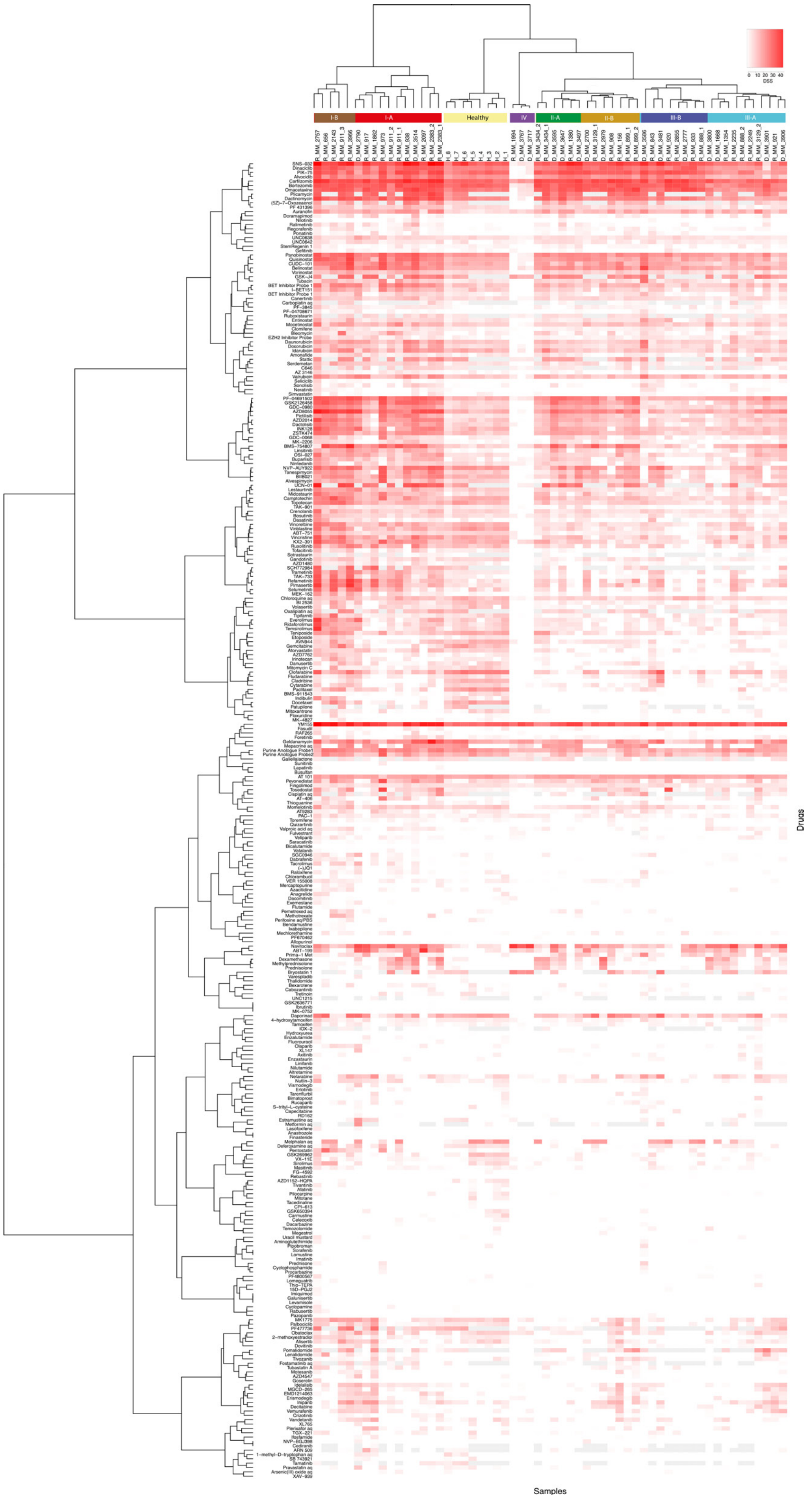

Supplementary Figure 1: Heatmap plotting drug sensitivity scores (DSS) for MM samples and healthy controls against 308 small molecule inhibitors. The heatmap was generated with drug sensitivity scores derived for each drug with CD138+ cells from 50 patient samples and 8 healthy controls. Clustering of drug sensitivity profiles was performed using unsupervised hierarchical ward-linkage clustering with Spearman correlation and Manhattan distance measures of drug response and sample profiles, respectively.

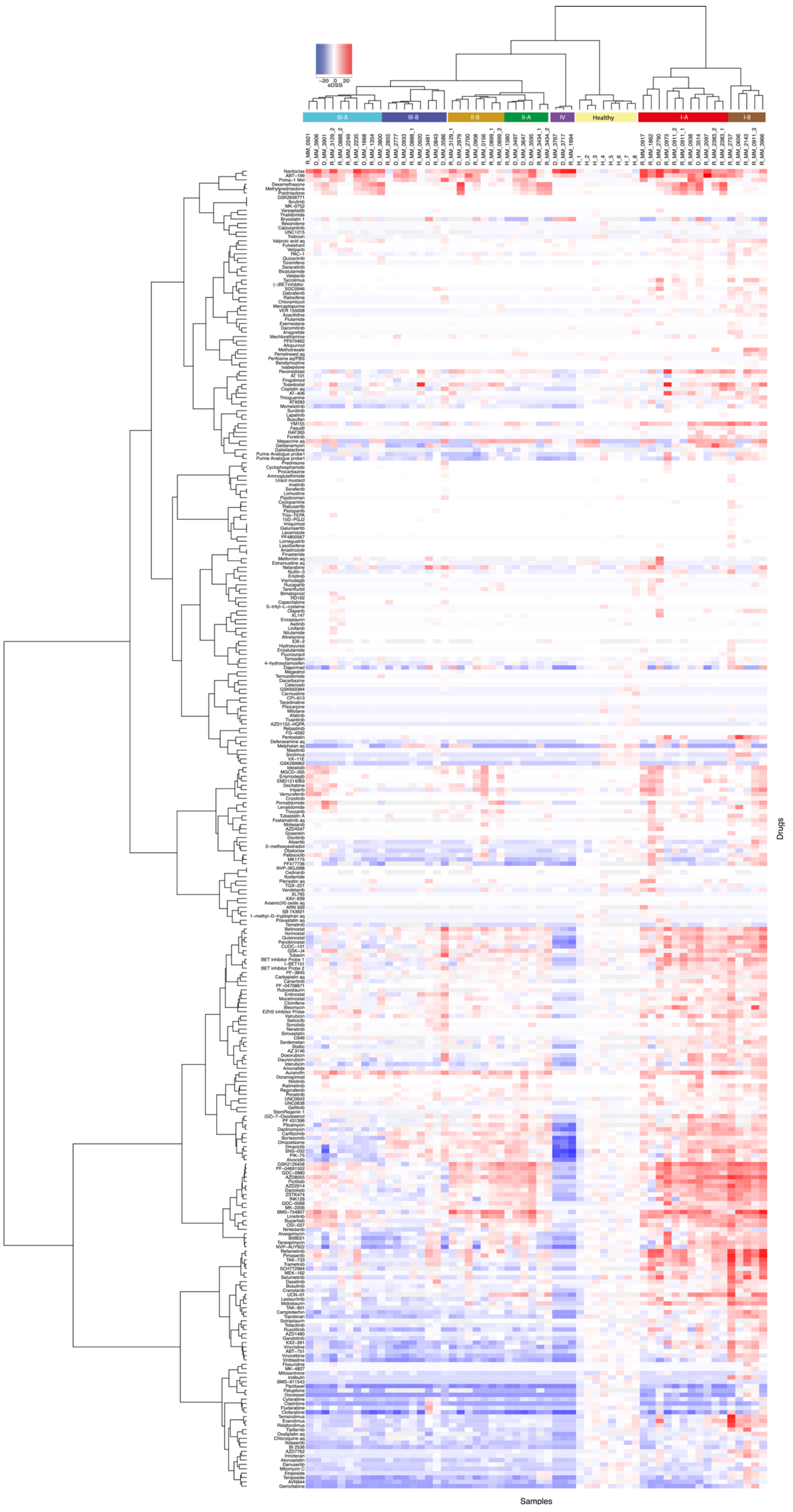

Supplementary Figure 2: Heatmap plotting selective drug sensitivity scores (sDSS) for 308 small molecules. The heatmap was generated with selective drug sensitivity scores derived from 50 patient samples and 8 healthy controls against the 308 inhibitors.

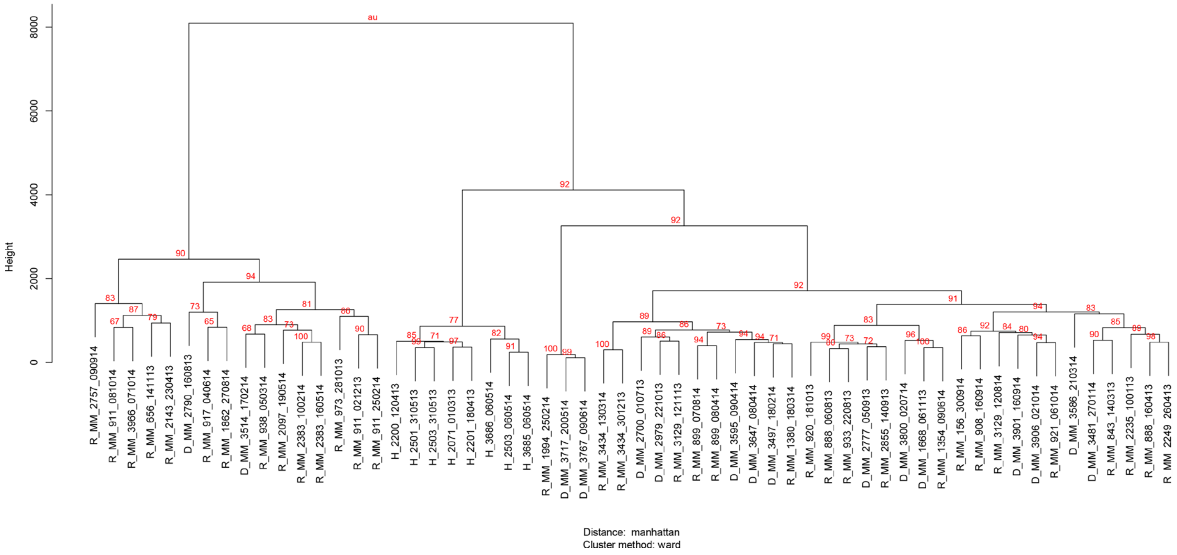

Supplementary Figure 3: Resampling by bootstrapping for evaluating the robustness of the observed clusters of myeloma patients. The DSS scores were bootstrapped (n = 1000) to assess the stability of the four drug response groups derived from cluster analysis. The results show the four major clusters can be reproduced with resampling.

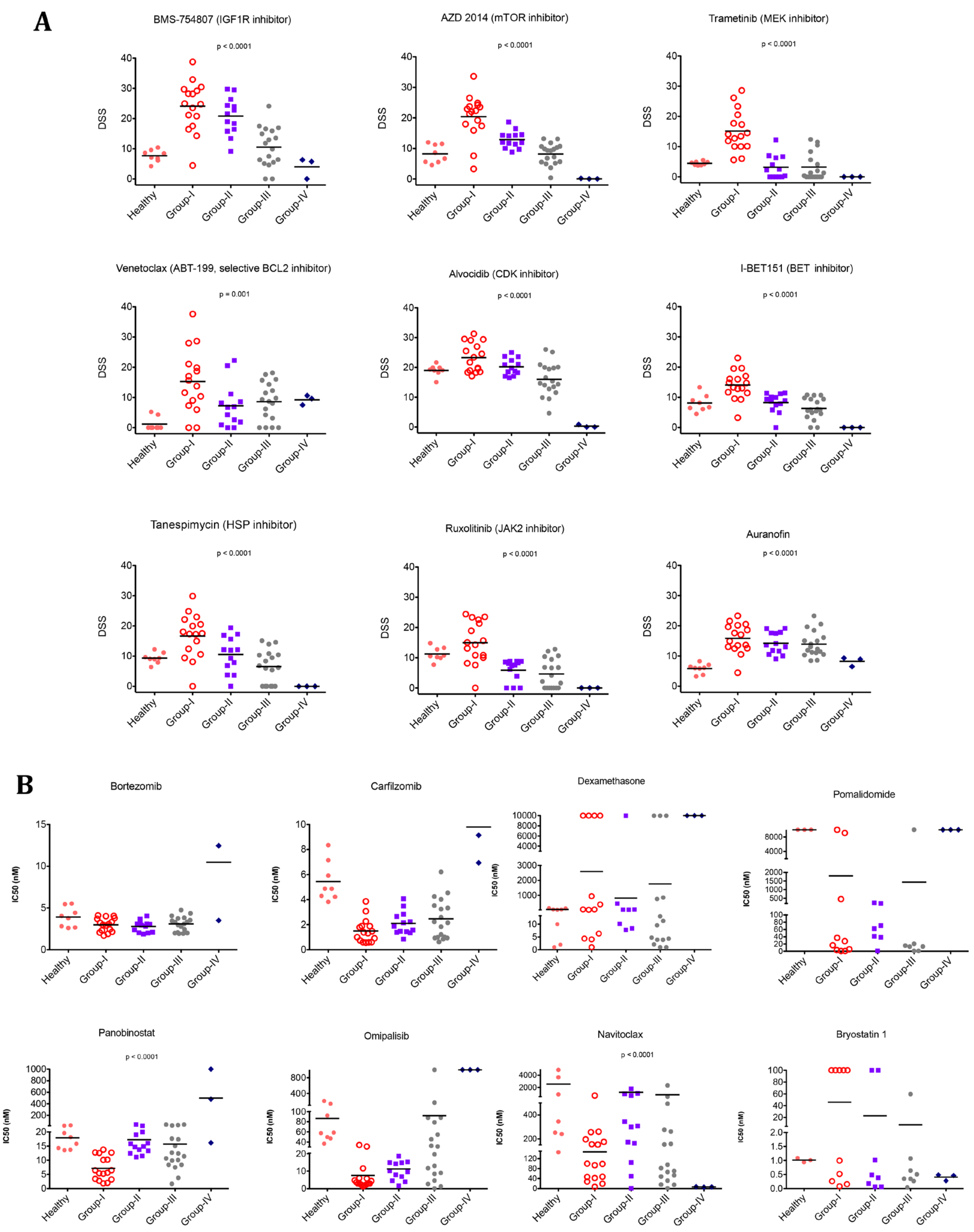

Supplementary Figure 4: Sensitivity of the MM CD138+ cells and healthy control cells to different targeted inhibitors. (**A**) The mean drug sensitivity scores (DSS) for the indicated inhibitors varied significantly between the four different drug response groups. (**B**) Graphs represent variation in IC50 among subgroups of patients for selected drugs presented in Figure 1B. Here, low IC50 values indicate better efficacy.

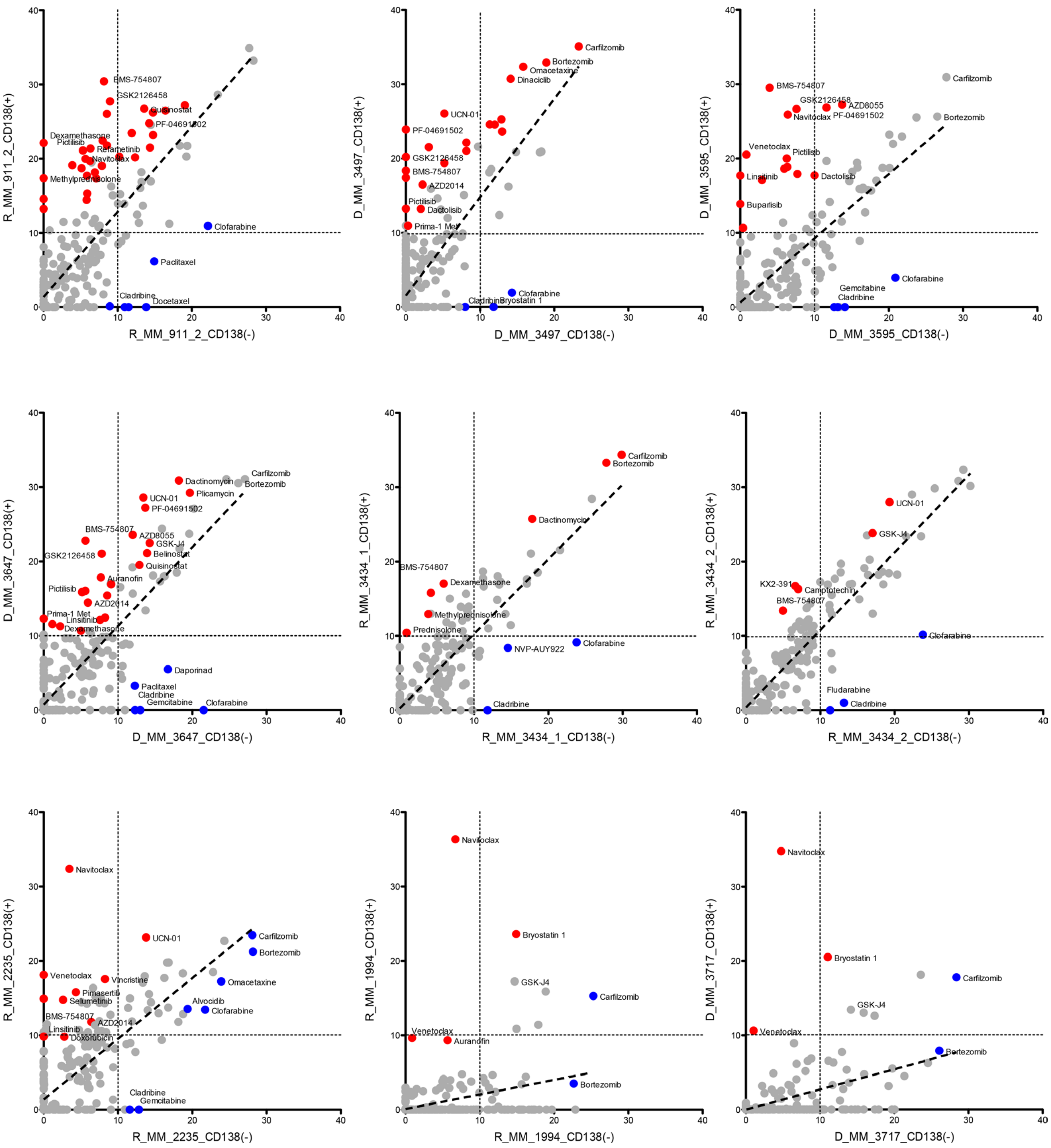

Supplementary Figure 5: Correlation plots showing the selective response pattern in CD138+ cells compared to CD138- cells from nine MM patients. Several small molecules were found to preferentially affect CD138+ cells (marked in red) compared to CD138- cells. CD138+ cells, however, were resistant to nucleoside analogues (marked in blue) compared to CD138- cells, which were sensitive to these drugs. Patients 1994 and 3717 are in the extremely resistant subgroup IV with CD138+ cells only exhibiting sensitivity to BCL2 inhibitors and bryostatin 1 (marked in red). Again, resistance was only seen in CD138+ cells compared to CD138- cells in those samples. In contrast, CD138- cells from these patients were sensitive to several drugs including proteasome inhibitors (marked in blue). Dashed lines indicate the cut-off values for sensitivity with drugs falling below a DSS value of 10 considered ineffective.

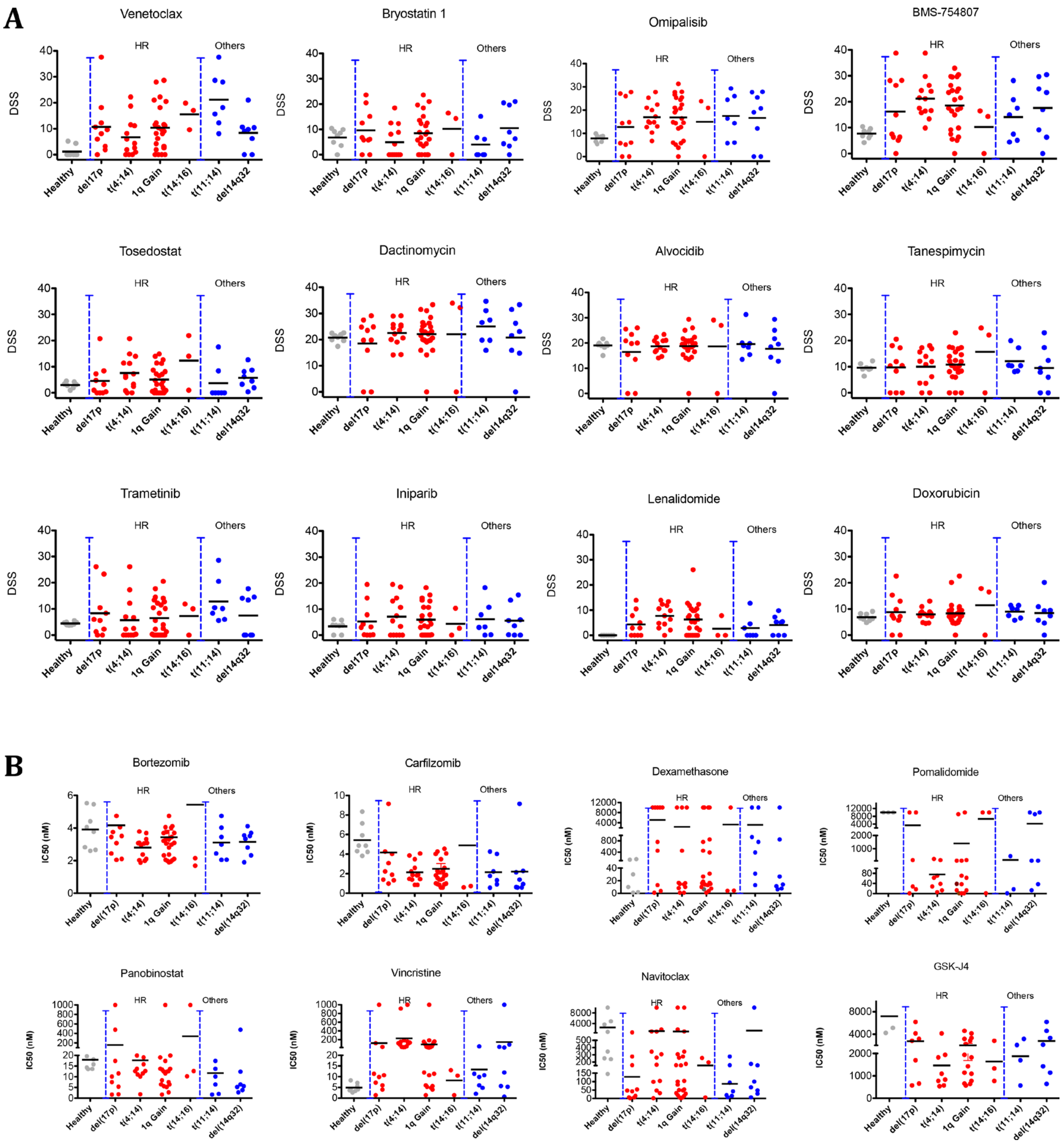

Supplementary Figure 6: The impact of cytogenetic lesions on drug response. (**A**) *Ex vivo* sensitivity of cells from patients with the indicated high risk (HR) and other cytogenetic alterations to drugs representing specific pharmacological modes of action. (**B**) Graphs showing IC50 values for the drugs presented in Figure 3B with samples grouped based on common cytogenetic alterations. Here, low IC50 values indicate better efficacy.

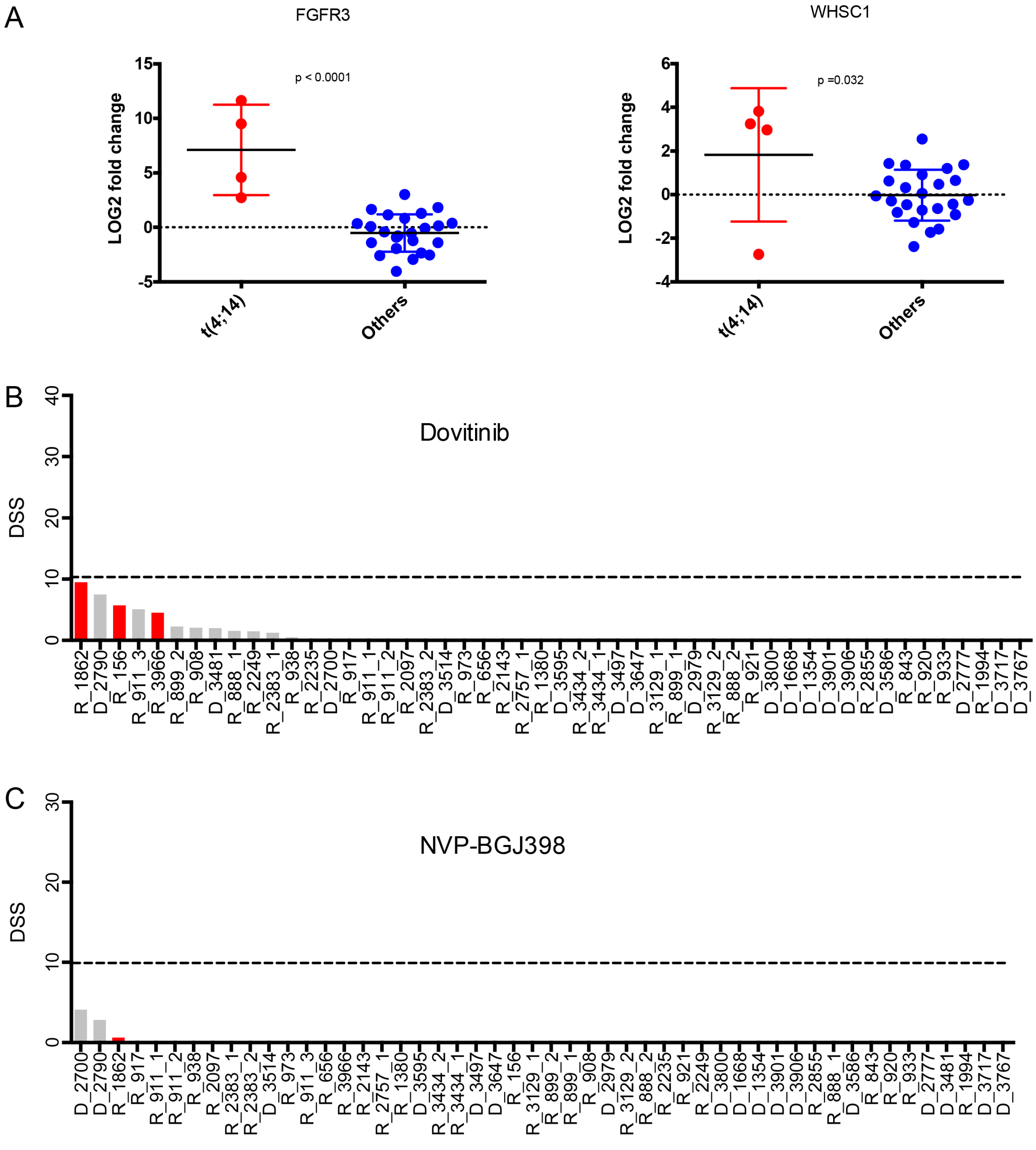

Supplementary Figure 7: The *FGFR3* and *WHSC1* (MMSET) genes are highly expressed in t(4;14) patients, but cells from t(4;14) patients are not sensitive to FGFR inhibitors. (**A**) Transcript levels of *FGFR3* and *WHSC1* are elevated in t(4;14) patient cells compared to other MM samples lacking the translocation. (**B**) *Ex vivo* sensitivity of all patient samples to the FGFR inhibitor dovotinib. (**C**) *Ex vivo* sensitivity to FGFR inhibitor NVP-BGJ938. The t(4;14) samples are indicated in red.

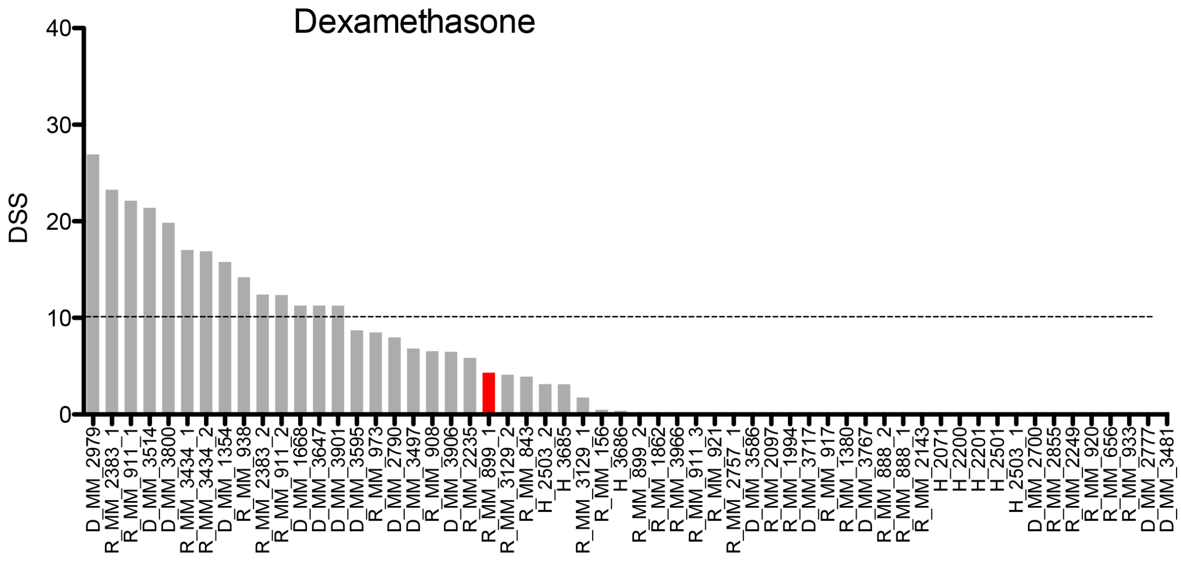

Supplementary Figure 8: ***Ex vivo* response to dexamethasone.** CD138+ cells from pomalidomide sensitive patients R_MM_899_2 and R_MM_1862 (indicated in red) were not sensitive to dexamethasone.

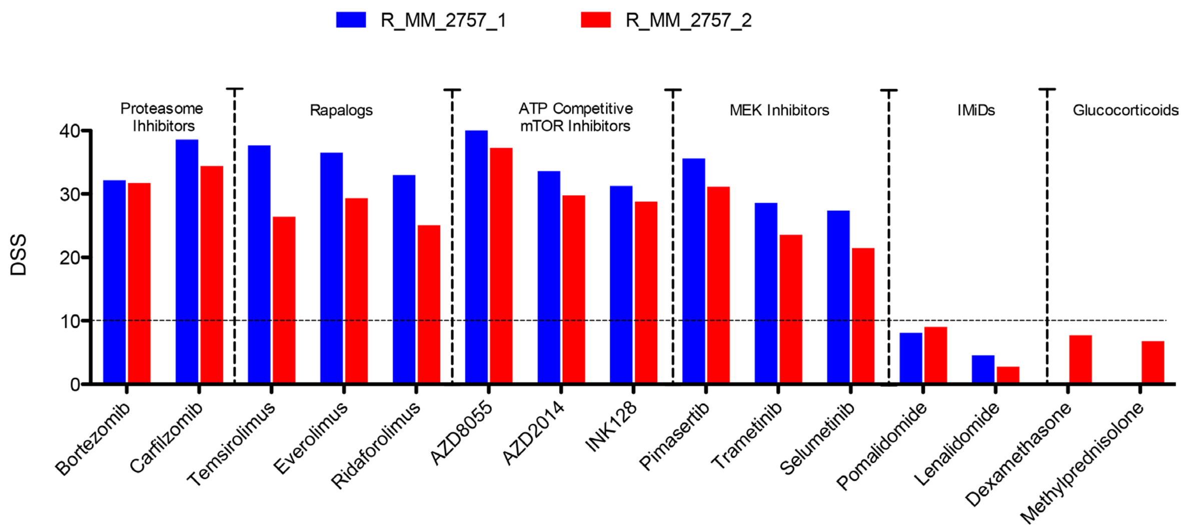

Supplementary Figure 9: ***Ex vivo* sensitivity of the pre- and post-temsirolimus treatment samples from patient R_MM_2757.** While there was a decrease in response to temsirolimus and other rapalogs in the post-treatment sample R_MM_2757_2 (red bars) compared to the pre-treatment sample (blue bars), only a modest change in sensitivity to ATP competitive mTOR inhibitors and MEK inhibitors was observed. Responses to proteasome inhibitors and immunomodulatory drugs were similar for the pre- and post-treatment samples, while an increase in sensitivity to glucocorticoids was observed in the post-treatment sample.
